# Chronoamperometric Observation and Analysis of Electrocatalytic Ability of Single Pd Nanoparticle for Hydrogen Peroxide Reduction Reaction

**DOI:** 10.3390/nano8110879

**Published:** 2018-10-26

**Authors:** June Young Park, Ki Jun Kim, Hyeryeon Son, Seong Jung Kwon

**Affiliations:** Department of Chemistry, Konkuk University, 120 Neungdong-ro Gwangjin-gu, Seoul 143-701, Korea; pa7673rk@naver.com (J.Y.P.); kim573252@naver.com (K.J.K.); envy5255@daum.net (H.S.)

**Keywords:** palladium, single nanoparticle, collision, electrocatalytic amplification, hydrogen peroxide

## Abstract

The current generated by the collision of a single nanoparticle (NP) of palladium (Pd) on a gold (Au) ultramicroelectrode (UME) surface was observed using an electrocatalytic amplification method. The hydrogen peroxide reduction reaction was used for the electrocatalytic reaction because the hydrogen peroxide reduction reaction has no gas-phase product, which would induce rapid signal decay. The electrocatalytic current resulting from a single Pd nanoparticle on the Au UME shows a staircase response with accompanying slow current decay. The applying potential and concentration of hydrogen peroxide were optimized for clear distinction of signal. The height of the current step and signal frequency were analyzed and compared with the theoretical expectation. The analysis of the electrocatalytic activity of single Pd NPs provides insight toward their future application.

## 1. Introduction

Advances in nanoscience and nanotechnology have given rise to the need to analyze nanomaterials at the level of a single nano-entity. The individual property of single nano-entity is more advisable than the ensemble-averaged properties of polydispersed system for the study of nanomaterial because the physicochemical properties of nanomaterials are very sensitive to their size, facet, surfactant, or supporter. However, the study of the electrocatalytic or other properties of a nanoparticle (NP) at the single NP level is highly challenging, because of the difficulty associated with identifying the weak signal among noise [1,2]. Recently, many electrochemistry groups have attempted to investigate the electrocatalytic properties of NPs at the single NP level using an electrochemical approach [3,4,5,6,7,8,9,10,11,12,13,14,15,16,17,18,19,20]. This technique, which is based on ampero- [3,4,5,6,7,8,9,10,11,12,13,14], potentio- [15,16], or coulo-metric [17] methods, exploits the electrocatalytic difference between an NP and a small electrode, such as an ultramicroelectrode (UME). 

To achieve this, an electrochemical cell consisting of an UME as the working electrode and an electrolyte solution containing the NPs is required. Whenever the NPs diffuse and collide on the UME, the change in electric signal at the moment of collision is observed. In this regard, the electrocatalytic amplification (EA) method [3,4,5,6,7,8,9,10,11,12,13,14,15,16,17,18,19,20], which was suggested by Bard group and is the most general method of this technique, was used for a highly electroactive metal or metal oxide NP. Whenever the active NPs collided on the inert UME, the electrocatalytic signal generated by an NP was recorded. A metallic NP with poor or no electrocatalytic activity can be detected by using direct particle electrolysis [6,18], which is based on the self-redox reaction of the NP itself. An insulating NP [19] (or soft particle), such as a nano-emulsion [20] or biomolecule [21,22], can be detected by using an UME blocking strategy [7,19] based on hindering the electrochemical reaction of the UME by the NP.

Noble metals, such as Pt, are widely studied by EA methods [3,4]. Even though Pt has good electrocatalytic activity for many useful electrochemical reactions, many researchers attempted to substitute it with a less expensive metal because of the limited natural reserves and high cost of Pt. One of the candidates to substitute Pt as an electrocatalyst is palladium (Pd). The versatile electrocatalytic activities of Pd have resulted in this metal being well studied as an electrocatalyst [23,24,25]. However, few studies at the single NP level have been reported for Pd NPs. Recently, Zare group reported firstly the collision of a single NP of Pd for the hydrazine oxidation reaction using a chronopotentiometric method [26].

In this study, we investigated the collision of a single Pd NP on an UME with chronoamperometric method using hydrogen peroxide reduction as the electrocatalytic reaction for EA. Here, hydrogen peroxide reduction [3,27,28] was selected instead of hydrazine oxidation [4], which is most widely used as the electrocatalytic reaction in experiments involving the collision of a single NP, for two reasons. First, the hydrogen peroxide reduction reaction does not have a gas-phase product. According to many previous studies, the current response of a single NP collision depends on various variables, including the electrochemical reaction product, resulting in a “blip” or “staircase” response [29]. When a gas-phase product is involved, the reaction is hindered by the adsorption of the produced gas on the surface of the NP, thereby inducing the decay of the current response. Therefore, the hydrogen peroxide reduction reaction, which can avoid this kind of current decay, was selected because the “staircase” response is much easier to analyze than the “blip” response. The second reason is the need to avoid excessive self-oxidation of the Pd NP. If we used an oxidation reaction, such as hydrazine oxidation, an anodic potential would be applied to the UME. This could affect the oxidation state of the NP and could ultimately change the electrocatalytic activity of the NP. Therefore, a reduction reaction is a much safer option for the self-oxidation of a Pd NP. In addition, the detection of hydrogen peroxide is important in biomedical and environmental applications.

Not only the shape of the current response, we also investigated the peak intensity and frequency whereby the Pd NP undergoes collisions. The shape corresponds to the mechanism or the change of the electrocatalytic ability of the Pd NP as a function of time. The peak intensity and frequency indicate the size and concentration of the Pd NP, respectively. The experimental values were recorded and analyzed by performing a theoretical calculation. In conclusion, the observation and analysis of the electrocatalytic activity of a single Pd NP for hydrogen peroxide reduction reaction using EA method can provide insight toward their application.

## 2. Materials and Methods 

### 2.1. Reagent

Palladium(II) chloride (PdCl_2_, 99%), sodium borohydride (NaBH_4_, ≥96%), potassium phosphate monobasic (KH_2_PO_4_, ≥99%), and citric acid (C_3_H_5_O(COOH)_3_, ≥99.5%) were obtained from Sigma-Aldrich (Aldrich, St. Louis, MO, USA). Hydrochloric acid (HCl, 35.0–37.0%), and hydrogen peroxide (H_2_O_2_, ~34.5%) were obtained from Samchun Pure Chemical Co., Ltd. (Pyeongtaek, Korea). Dipotassium hydrogen phosphate (K_2_HPO_4_, 99.0%) was obtained from Junsei Chemical Co., Ltd. (Tokyo, Japan). All chemicals were used as received. Ultrapure water (≥18 MΩ, Millipore, Bedford, MA, USA) was used in all experiments.

### 2.2. Preparation of Pd NP

An aqueous solution of 0.5 mM of PdCl_2_ was prepared by completely dissolving 1.773 mg of PdCl_2_ in 20 mL of 1 mM HCl under ultrasonic treatment for 30 min. After that, 3.842 mg of citric acid (1 mM) was dissolved in the mixed solution with magnetic stirring for 5 min at 80 °C and 2 mL of 0.1 M NaBH_4_ aqueous solution was slowly added to the mixture under magnetic stirring. The color of the solution became dark brown [30].

### 2.3. Preparation of UME

The Pd and Au UMEs were prepared using the following method. Briefly, Pd wire with a 10 µm radius or Au wire with a 6.35 µm radius was sealed in glass after rinsing with methanol and water. The electrode was then polished with alumina powder water suspension to a mirror face. The surface area was checked with standard redox electrochemistry of ferrocene in methanol [3,4,5].

### 2.4. Electrochemical Cell and Technique

All experiments were conducted in a conventional three-electrode cell at room temperature. The working electrode was the UME. A platinum electrode was used as the counter electrode and a standard Ag/AgCl electrode as the reference electrode. All electrochemical experiments were performed in 0.1 M phosphate buffer (PB) solutions. The cyclic voltammetry was done at a 100 mV/s scan rate in pH 13 solution. The chronoamperometric curves were obtained with 50 ms of data acquisition time. The NP was injected into the electrolyte solution after starting the chronoamperometric measurement usually 10 s later.

### 2.5. Instrumentation

Electrochemical experiments were performed with a CHI 660A electrochemical analyzer (CH Instruments, Austin, TX, USA). The pH of the solution was measured by a EZRODE P1 (EZSEN, Seoul, Korea). The transmission electron microscopy (TEM) image was obtained from the Korea Basic Science Institute (Seoul, Korea) using a Tecnai G2 F30ST Instrument (FEI Company, Hillsboro, OR, USA). 

## 3. Results and Discussion

**Figure 1:**
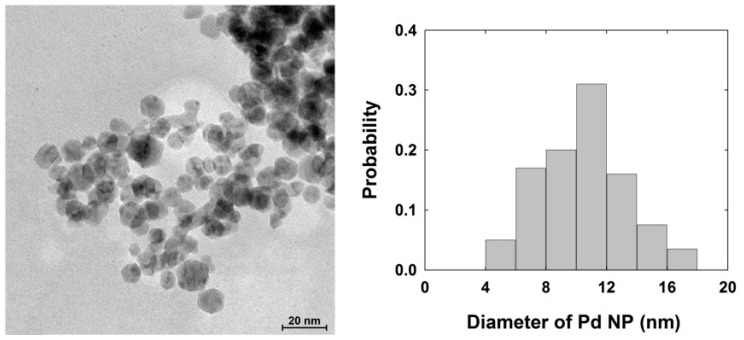
Transmission electron microscopy (TEM) image of palladium (Pd) nanoparticle (NP) and its size distribution. The scale bar is 20 nm.

The size of Pd nanoparticles (NPs) was determined by acquiring TEM images as shown in Figure 1. The average diameter of Pd NPs was approximately 11 (±3) nm. The concentration of stock solution of Pd NPs was calculated from the concentration of the Pd precursor divided by the average number of Pd atoms contained in a particle. The average number of Pd atoms in a 11 nm sized Pd NP was estimated as ~4.74 × 10^4^ atoms. The average number of atoms per particle was calculated by the proportional basis of density and radius between single atom and NP.

Electrocatalytic amplification (EA) methods are designed to detect collisions of a NP on an inert electrode. The observation of the current response of a single NP collision depends on the kind of NPs and the ultramicroelectrode (UME). Even the same NP sometimes shows different signal responses for the collision with different UMEs. This is because the adsorption behavior between the NP and UME is very sensitive to the materials of which the NP and UME consist. We found the Pd NP on Au electrode to show good electrocatalytic activity for hydrogen peroxide reduction. Therefore, an Au UME was selected as inert electrode to observe the collision of the Pd NP.

**Figure 2:**
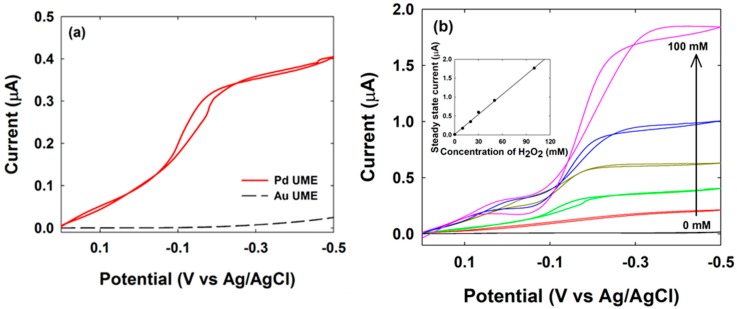
Cyclic voltammograms of hydrogen peroxide reduction reaction at: (**a**) Au (black dashed) or Pd (red solid) ultramicroelectrode (UME) (radius 6.35 and 10 µm, respectively) in a 0.1 M phosphate buffer (PB) solution (pH 6.8) containing 20 mM H_2_O_2_; (**b**) Pd UME in 0.1 M PB solution (pH 6.8) containing 0, 10, 20, 30, 50, and 100 mM H_2_O_2_. Scan rate was 100 mV/s.

Next, we investigated the electrocatalytic ability of both an Au and a Pd UME for the hydrogen peroxide reduction to determine an appropriate potential adaptable for the electrocatalytic reaction by only the NP excluding the UME. As shown in Figure 2a, the Au and Pd UMEs exhibited different behavior for the electrochemical reduction of hydrogen peroxide. At the Pd UME, the onset potential of the hydrogen peroxide reduction reaction is less negative than that at the Au UME. As expected, the Pd UME showed considerable current even though the Au UME has low electrocatalytic activity for the hydrogen peroxide reduction. To confirm that this reduction current originated from the hydrogen peroxide reduction rather than proton reduction or other reactions, the change in the reduction current at the Pd UME was investigated at various hydrogen peroxide concentrations from 0 to 100 mM. As shown in Figure 2b, the steady-state current increased proportional to the concentration of hydrogen peroxide. Therefore, the main contribution to the reduction current is by hydrogen peroxide reduction in this potential window and at this pH. However, the potential range for which a steady-state current exists at the Pd UME is not leveled and the background current at the Au UME also increases slightly below −0.2 V as shown in Figure 2 and Figure S1 (see the Supporting Information, SI). This might be a contribution from the proton reduction reaction or other reactions. When the collision current generated by the NP was recorded at −0.2 V or more negative values by chronoamperometry, the background current fluctuated considerably (Figure S2). The regular pattern of current fluctuation was obtained at this case, which seems due to the regular bubble production by the side reaction such as hydrogen evolution [31]. Thus, it was difficult to distinguish the signal from the noise. This prompted us to select −0.15 V at which to conduct the chronoamperometric experiment to observe the collision of a single NP with the aim of minimizing the contribution of side reactions to the background current. 

Similarly, the signal distribution from the noise background current was more problematic at higher concentrations of hydrogen peroxide. Therefore, the hydrogen peroxide concentration was optimized to 20 mM (Figure S3).

**Figure 3:**
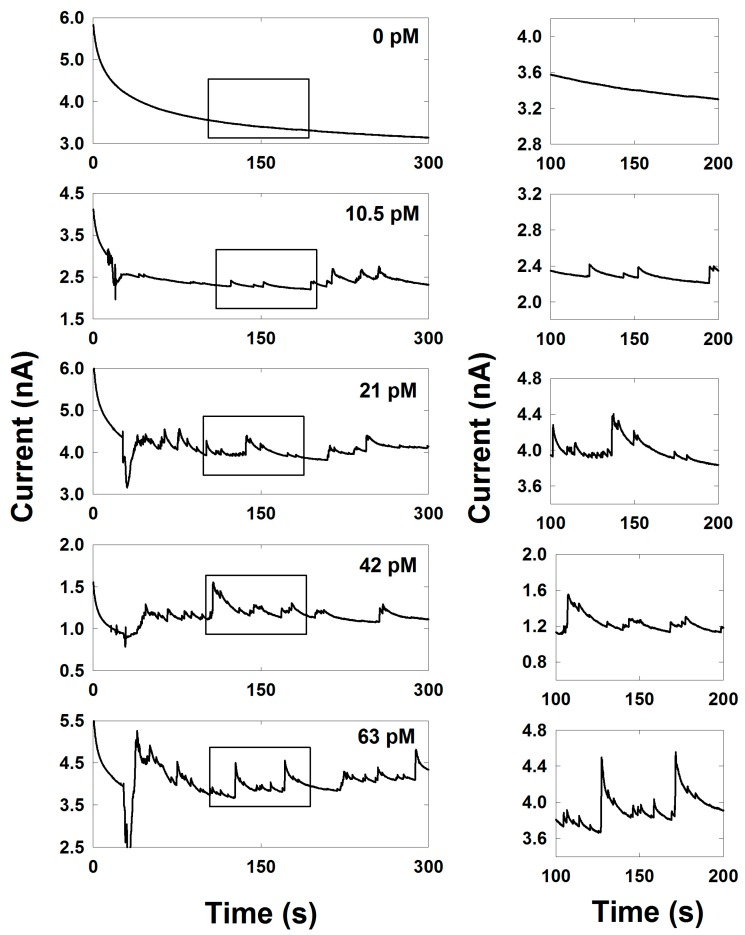
Chronoamperometric curves for single Pd NP collisions at an applied potential of −0.15 V at the Au UME with different Pd NP concentrations (0, 10.5, 21, 42, and 63 pM) in a 0.1 M PB solution containing 20 mM H_2_O_2_. The data acquisition time was 50 ms.

The electrocatalytic activity of a single Pd NP was investigated by obtaining the chronoamperometric measurement as shown in Figure 3. The collision of the Pd NP with the Au UME, held at a potential of −0.15 V, led to the observation of a staircase-like response of the current transient. However, the instant increase in the current is not maintained at this level and shows a very slow decay.

In many previous studies, relatively fast decay, resulting in a “blip” response, was observed when the single NP collision system consisted of an electrocatalytic reaction with a gas-phase product. The Pt NP/Ni UME/hydrazine oxidation system exhibits a current response change from “staircase” to “blip” under specific conditions [29]. Likewise, the IrO_x_ NP/Pt UME/water oxidation system has a “blip” response due to the oxygen gas product that forms an enclosure on the surface of the NP [32]. Therefore, these gas-phase products, such as nitrogen in hydrazine oxidation or oxygen in water oxidation, are responsible for the “blip” response by attaching to the surface of the NP and preventing the NPs from participating in electron transfer. However, in this system, the only reaction product of hydrogen peroxide reduction is water. Therefore, there seems to be another reason for the deactivation of the Pd NP as manifested by the very slow decay of the current response, such as the adsorption of impurities or a change in the state of the NP or by the gas-phase product of side reaction such as proton reduction.

In previous studies, collision experiments with the Pd NP using the hydrazine oxidation reaction showed a “blip” response [26]. However, when the hydrogen peroxide reduction was used with a Pt NP, a “staircase” response was obtained similar to our result [3]. When hydrogen peroxide oxidation was used for the detection of a RuO_x_ NP, the signal showed the “blip” response due to the gas-phase product oxygen [27]. Therefore, single-NP detection was accomplished by using hydrogen peroxide reduction, which causes the “staircase” response because of the absence of a gas-phase product. 

**Figure 4:**
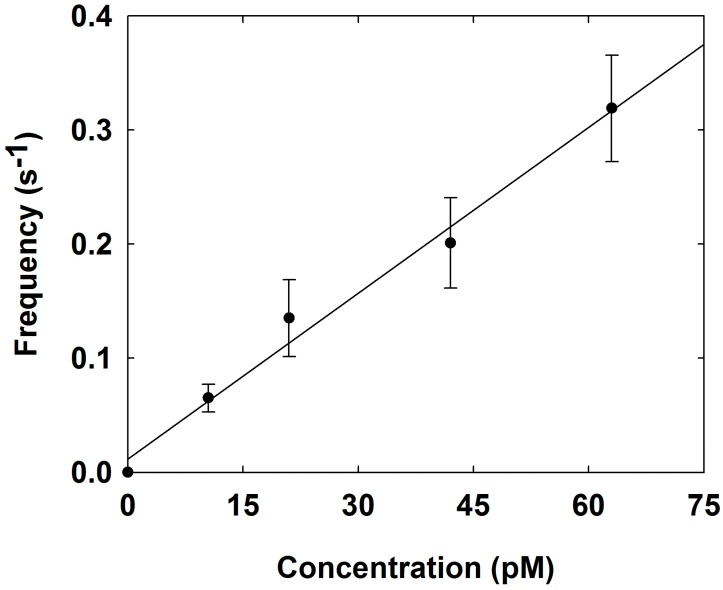
Collision frequency as a function of Pd NP concentration at an applied potential of −0.15 V at the Au UME in a 0.1 M PB solution containing 20 mM H_2_O_2_ (for 4 replicate measurements).

The collision frequencies were investigated in the presence of various concentrations (0, 10.5, 21, 42, and 63 pM) of Pd NPs. The frequency was proportional to the concentration of the NPs as expected and as shown in Figure 3 and Figure 4. The experimentally obtained collision frequency was 0.0048 s^−1^ pM^−1^. However, the collision frequency was approximately three orders of magnitude lower than the value (0.681 s^−1^ pM^−1^) calculated by the Fick’s law [33,34]:

$$f_{p}{\;=\;4\;D}_{\mathrm{NP}}{\;C}_{\mathrm{NP}}\;r_{\mathrm{UME}}\;$$

where $C_{\mathrm{NP}}$ is the concentration of NPs, $r_{\mathrm{UME}}$ is the radius of the UME, $D_{\mathrm{NP}}$ is the diffusion coefficient of NP, which is estimated as 4.46 × 10^−7^ cm^2^ s^−1^ by the Einstein-Stokes equation. The lower frequency originates from the aggregation of Pd NPs in electrolyte solution, the loss of NPs by adherence to the cell wall or precipitate, or loss of signal by noisy background current or a lower adsorption coefficient between Pd NPs and the Au UME. 

The frequency and the peak intensity of the current signal were investigated. The theoretical steady-state current value by NP, $I_{\mathrm{SS},\mathrm{NP}}$, could be calculated by the following equation [4]:

$$\;I_{\mathrm{SS},\mathrm{NP}}{\;=\;4\pi \;\ln2\;n\;F\;D}_{H_{2}O_{2}}C_{H_{2}O_{2}}r_{\mathrm{NP}}\;$$

where *n* is the number of electrons, *F* is the Faraday coefficient, $D_{H_{2}O_{2}}$ is the diffusion coefficient of hydrogen peroxide, $C_{H_{2}O_{2}}$ is the concentration of hydrogen peroxide, and $r_{\mathrm{NP}}$ is the radius of the NP. Here, the diffusion coefficient of hydrogen peroxide, $D_{H_{2}O_{2}}$, is estimated by following equation [34]:

$$\;I_{\mathrm{SS},\mathrm{UME}}{\;=\;4\;n\;F\;D}_{H_{2}O_{2}}C_{H_{2}O_{2}}r_{\mathrm{UME}}\;$$

where $I_{\mathrm{SS},\mathrm{UME}}$ is the steady-state current of the UME, $r_{\mathrm{UME}}$ is the radius of the UME. The diffusion coefficient, 2.59 × 10^−5^ cm^2^ s^−1^, was obtained from the Figure 1 using a steady-state current of 0.6 µA at the 30 mM of hydrogen peroxide concentration, a 10 µm radius of the Pd UME, and a two-electron transfer reaction. 

As a result of the calculation above, the theoretical steady-state current by single Pd NP was 479 pA. However, the experimentally applied potential, −0.15 V, is not the potential for steady-state region. It is slightly lower than the steady-state value. Therefore, we multiplied a ratio factor, 0.83, to obtain the final estimated current, 399 pA, which is the expected current at −0.15 V where the chronoamperometric measurement was done to prevent background current fluctuation. The experimentally obtained current steps ranged from 20 to 600 pA with average value of 110 (±90) pA (Figure S4), which is of the same order of magnitude as the theoretical value. We didn’t count the current step below 20 pA, because it is difficult to distinguish from noise. The relatively smaller experimental current step compared to the calculation may be due to the lower electrocatalytic activity of Pd NP on Au UME, competition with other reactions, or aggregation of NPs. If the NP became bigger by aggregation, the diffusion coefficient is decreased. Therefore, the collision probability of bigger particle during the experimental time domain, ~300 s, is decreased, so the contribution by small particle is dominant at a short time domain.

## 4. Conclusions

We have investigated the electrocatalytic activity of a single Pd NP for hydrogen peroxide reduction reaction by observing the collision of NP on the Au UME using EA method. The collision event of a single Pd NP was successfully recorded as a staircase current transient with accompanying slow current decay. The hydrogen peroxide reduction has no gas-phase product, the slow decay indicated the deactivation of Pd NP on the Au UME for the hydrogen peroxide reduction. The magnitude of the current generated by the collisions of the NP represents the size distribution of NPs, and the collision frequency is directly proportional to the concentration of the Pd NPs. This observation and analysis of single NP can be used for the identification of a high performance nanocatalyst from numerous NPs or a sensing scheme of ultrasensitive biosensor by employing the nanoparticle and the EA methods as a label and detection system.

## Supplementary Materials

The following are available online at http://www.mdpi.com/2079-4991/8/11/879/s1, Figure S1: Cyclic voltammograms of background reaction at Au (black dashed) or Pd (red solid) UME (radius 6.35 and 10 µm, respectively) in a 0.1 M PB solution (pH 6.8) without H_2_O_2_, Scan rate was 100 mV/s. Figure S2: Chronoamperometric curves for single Pd NP collisions at an applied potential of −0.3 V (top) or −0.2 V (bottom) at the Au UME with 10.5 pM of Pd NP concentrations in a 0.1 M PB solution containing 10 mM H_2_O_2_. The data acquisition time was 50 ms. Figure S3: Chronoamperometric curves for single Pd NP collisions at −0.15 V applied the Au UME with 10.5 pM of Pd NP concentrations in a 0.1 M PB solution containing various H_2_O_2_ concentrations (10 mM, 20 mM, and 30 mM). Figure S4: Distribution of current height of single Pd NP collision. Figure S5: TEM image of Pd NP and its energy-dispersive X-ray spectroscopy (EDX) analysis. The scale bar is 500 nm. Figure S6: Photo of electrochemical set-up its schematic illustration.

## References

[1] Sönnichsen C., Reinhard B.M., Liphardt J., Alivisatos P. (2005). A molecular ruler based on plasmon coupling of single gold and silver nanoparticles. Nat. Biotechnol..

[2] Fan F.-R.F., Bard A.J. (1997). An electrochemical coulomb staircase: Detection of single electron-transfer events at nanometer electrodes. Science.

[3] Xiao X., Bard A.J. (2007). Observing single nanoparticle collisions at an ultramicroelectrode by electrocatalytic amplification. J. Am. Chem. Soc..

[4] Xiao X., Fan F.-R.F., Zhou J., Bard A.J. (2008). Current transient in single nanoparticle collision events. J. Am. Chem. Soc..

[5] Kwon S.J., Fan F.-R.F., Bard A.J. (2010). Observing Iridium oxide (IrO_x_) single nanoparticle collisions at ultramicroelectrodes. J. Am. Chem. Soc..

[6] Zhou Y.-G., Rees N.V., Compton R.G. (2011). The electrochemical detection and characterization of silver nanoparticles in aqueous solution. Angew. Chem. Int. Ed..

[7] Quinn B.M., van’t Hof P.G., Lemay S.G. (2004). Time-resolved electrochemical detection of discrete adsorption events. J. Am. Chem. Soc..

[8] Kleijn S.E.F., Lai S.C.S., Miller T.S., Yanson A.I., Koper M.T.M., Unwin P.R. (2012). Landing and catalytic characterization of individual nanoparticles on electrode surfaces. J. Am. Chem. Soc..

[9] Fosdick S.E., Anderson M.J., Nettleton E.G., Crooks R.M. (2013). Correlated electrochemical and optical tracking of discrete collision events. J. Am. Chem. Soc..

[10] Dasari R., Robinson D.A., Stevenson K.J. (2013). Ultrasensitive electroanalytical tool for detecting, sizing, and evaluating the catalytic activity of platinum nanoparticles. J. Am. Chem. Soc..

[11] Guo Z.-H., Percival S.J., Zhang B. (2014). Chemically resolved transient collision events of single electrocatalytic nanoparticles. J. Am. Chem. Soc..

[12] Fernando A., Parajuli F., Alpuche-Aviles M.A. (2013). Observation of individual semiconducting nanoparticle collisions by stochastic photoelectrochemical currents. J. Am. Chem. Soc..

[13] Chen C.-H., Ravenhill E.R., Momotenko D., Kim Y.-R., Lai S.C.S., Unwin P.R. (2015). Impact of surface chemistry on nanoparticle-electrode interactions in the electrochemical detection of nanoparticle collisions. Langmuir.

[14] Ustarroz J., Kang M., Bullions E., Unwin P.R. (2017). Impact and oxidation of single silver nanoparticles at electrode surface: One shot versus multiple events. Chem. Sci..

[15] Zhou H., Park J.H., Fan F.-R.F., Bard A.J. (2012). Observation of single metal nanoparticle collisions by open circuit (mixed) potential changes at an ultramircoelectrode. J. Am. Chem. Soc..

[16] Dasari R., Robinson D.A., Stevenson K.J. (2014). Electrochemical Monitoring of Single Nanoparticle Collisions at Mercury-Modified Platinum Ultramicroelectrodes. ACS Nano.

[17] Zhou Y.-G., Rees N.V., Pillay J., Tshikhudo R., Vilakazi S., Compton R.G. (2012). Gold nanoparticles show electroactivity: Counting and sorting nanoparticles upon impact with electrodes. Chem. Commun..

[18] Haddou B., Rees N.V., Compton R.G. (2012). Nanoparticle-electrode impacts: The oxidation of copper nanoparticles has slow kinetics. Phys. Chem. Chem. Phys..

[19] Boika A., Thorgaard S.N., Bard A.J. (2013). Monitoring the electrophoretic migration and adsorption of single insulating nanoparticles at ultramicroelectrodes. J. Phys. Chem. B.

[20] Kim B.K., Kim J., Bard A.J. (2015). Electrochemistry of a single attoliter emulsion droplet in collisions. J. Am. Chem. Soc..

[21] Dick J.E., Hilterbrand A.T., Boika A., Upton J.W., Bard A.J. (2015). Electrochemical detection of single cytomegalovirus at an ultramicroelectrode and its antibody anchoring. Proc. Natl. Acad. Sci. USA.

[22] Dick J.E., Renault C., Bard A.J. (2015). Obeservation of single-protein and DNA macromolecule collisions on ultramicroelectrodes. J. Am. Chem. Soc..

[23] Safavi A., Maleki N., Tajabadi F., Farjami E. (2007). High electrocatalytic effect of palladium nanoparticle arrays electrodeposited on carbon ionic liquid electrode. Electrochem. Commun..

[24] Chen X.-M., Cai Z.-X., Huang Z.-Y., Oyama M., Jiang Y.-Q., Chen X. (2013). Ultrafine palladium nanoparticles grown on graphene nanosheets for enhanced electrochemical sensing of hydrogen peroxide. Electrochim. Acta.

[25] You J.-M., Jeong Y.N., Ahmed M.S., Kim S.K., Choi H.C., Jeon S. (2011). Reductive determination of hydrogen peroxide with MWCNTs-Pd nanoparticles on a modified glassy carbon electrode. Biosens. Bioelectron..

[26] Daryanavard N., Zare H.R. (2017). Single Palladium nanoparticle collisions detection through chronopotentiometric method: Introducing a new approach to improve the snalytical signals. Anal. Chem..

[27] Kang M., Perry D., Kim Y.-R., Colburn A.W., Lazenby R.A., Unwin P.R. (2015). Time-resolved detection and analysis of single nanoparticle electrocatalytic impacts. J. Am. Chem. Soc..

[28] Stuart E.J.E., Rees N.V., Compton R.G. (2012). Particle-impact voltammetry: The reduction of hydrogen peroxide at silver nanoparticles impacting a carbon electrode. Chem. Phys. Lett..

[29] Jung A.R., Lee S., Joo J.W., Shin C., Bae H., Moon S.G., Kwon S.J. (2015). Potential-controlled current responses from staircase to blip in single Pt nanoparticle collisions on a Ni ultramicroelectrode. J. Am. Chem. Soc..

[30] Guo Y., Xu Y.-T., Gao G.-H., Wang T., Zhao B., Fu X.-Z., Sung R., Wong C.-P. (2015). Electro-oxidation of formaldehyde and methanol over hollow porous palladium nanoparticles with enhanced catalytic activity. Catal. Commun..

[31] Kim J.J., Choi Y.S., Kwon S.J. (2013). Study on Electrocatalytic water oxidation reaction by Iridium oxide and its bubble overpotential Effect. J. Korean Electrochem. Soc..

[32] Kwon S.J., Bard A.J. (2012). Analysis of diffusion-controlled stochastic events of iridium oxide single nanoparticle collisions by scanning electrochemical microscopy. J. Am. Chem. Soc..

[33] Kwon S.J., Zhou H., Fan F.-R.F., Vorobyev V., Zhang B., Bard A.J. (2011). Stochastic electrochemistry with electrocatalytic nanoparticles at inert ultramicroelectrodes—Theory and experiments. Phys. Chem. Chem. Phys..

[34] Bard A.J., Faulkner L.R. (2001). Ectrochemical Methods, Fundamentals and Applications.

