# Supplementary material for: Chronoamperometric Observation and Analysis of Electrocatalytic Ability of Single Pd Nanoparticle for Hydrogen Peroxide Reduction Reaction
Source: Nanomaterials (Basel). 2018 Oct 26;8(11):879. doi: 10.3390/nano8110879 (PMC6266670; doi:10.3390/nano8110879)
Supplement: Supplementary file 1 [file nanomaterials-08-00879-s001.pdf]

Supporting Information

Chronoamperometric Observation and Analysis of Electrocatalytic Ability of Single Pd Nanoparticle for Hydrogen Peroxide Reduction Reaction

June Young Park, Ki Jun Kim, Hyeryeon Son and Seong Jung Kwon*

Department of Chemistry, Konkuk University, 120 Neungdong-ro Gwangjin-gu, Seoul 143-701, Korea; pa7673rk@naver.com (J.Y.P.); kim573252@naver.com (K.J.K.); envy5255@daum.net (H.S.)

* Correspondence: sjkwon@konkuk.ac.kr; Tel.: +82-2-450-0429

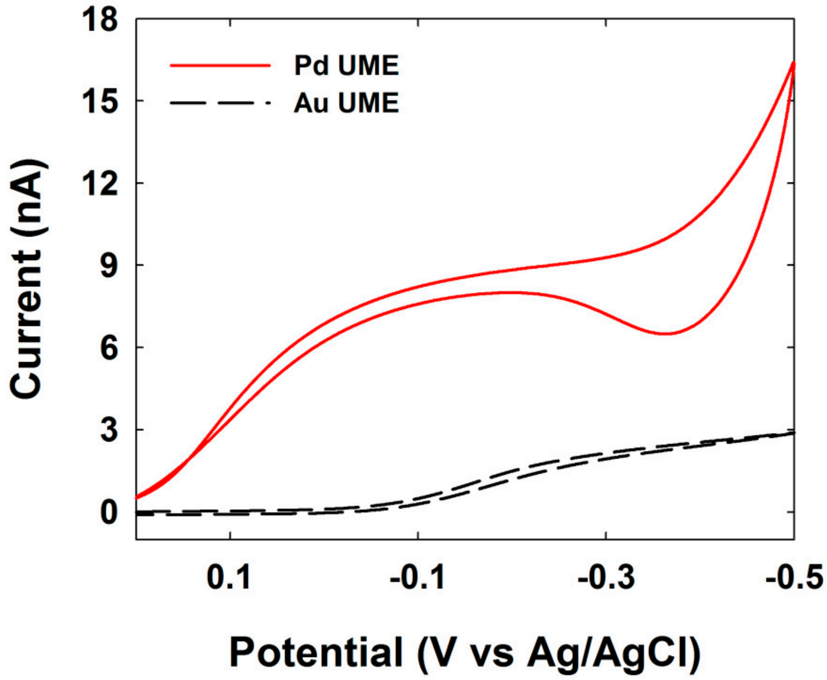

Figure S1. Cyclic voltammograms of background reaction at Au (black dashed) or Pd (red solid) UME (radius 6.35 and 10 μm, respectively) in a 0.1 M PB solution (pH 6.8) without H_2O_2, Scan rate was 100 mV/s.

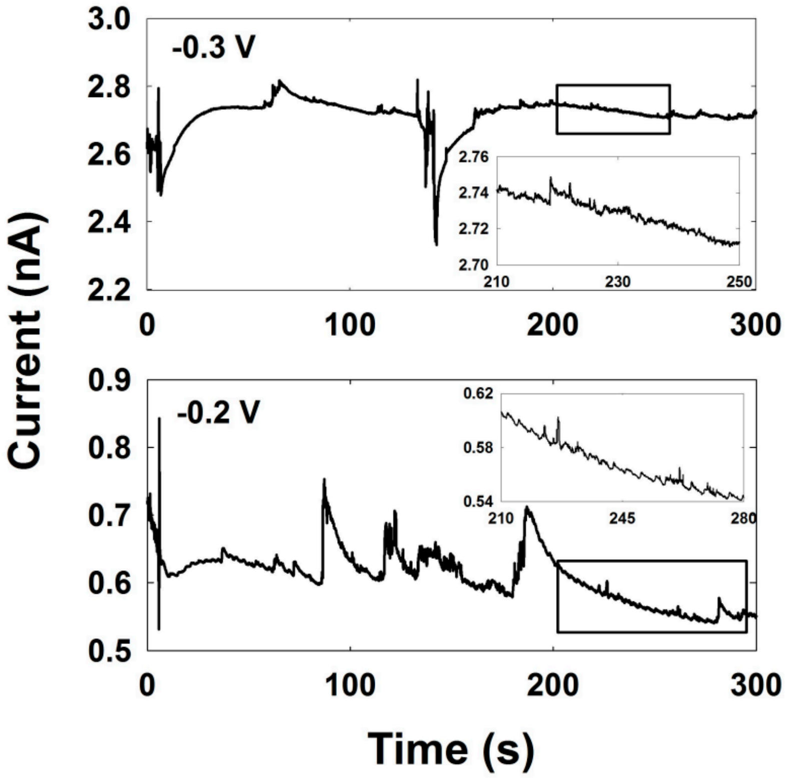

Figure S2. Chronoamperometric curves for single Pd NP collisions at an applied potential of –0.3 V (top_ or –0.2 V (bottom) at the Au UME with 10.5 pM of Pd NP concentrations in a 0.1 M PB solution containing 10 mM H_2O_2. The data acquisition time was 50 ms.

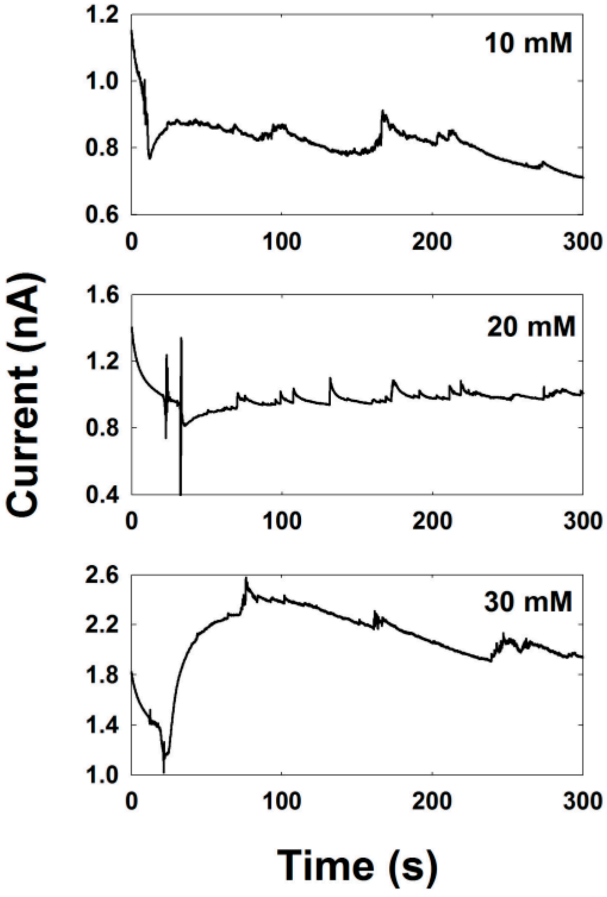

Figure S3. Chronoamperometric curves for single Pd NP collisions at -0.15 V applied the Au UME with 10.5 pM of Pd NP concentrations in a 0.1 M PB solution containing various H_2O_2 concentrations (10 mM, 20 mM, and 30 mM).

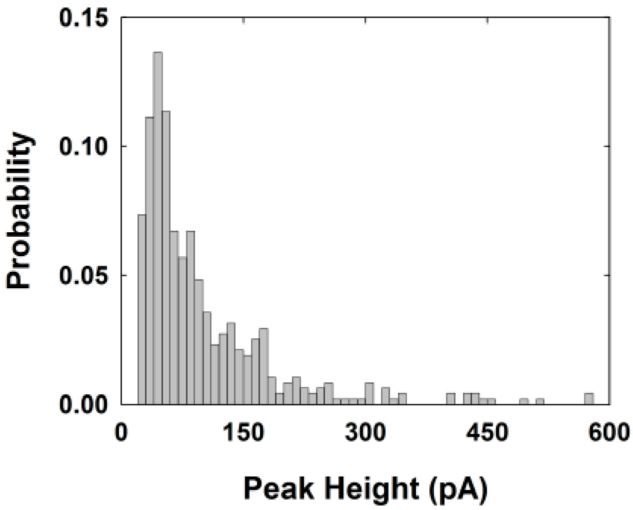

Figure S4. Distribution of current height of single Pd NP collision.

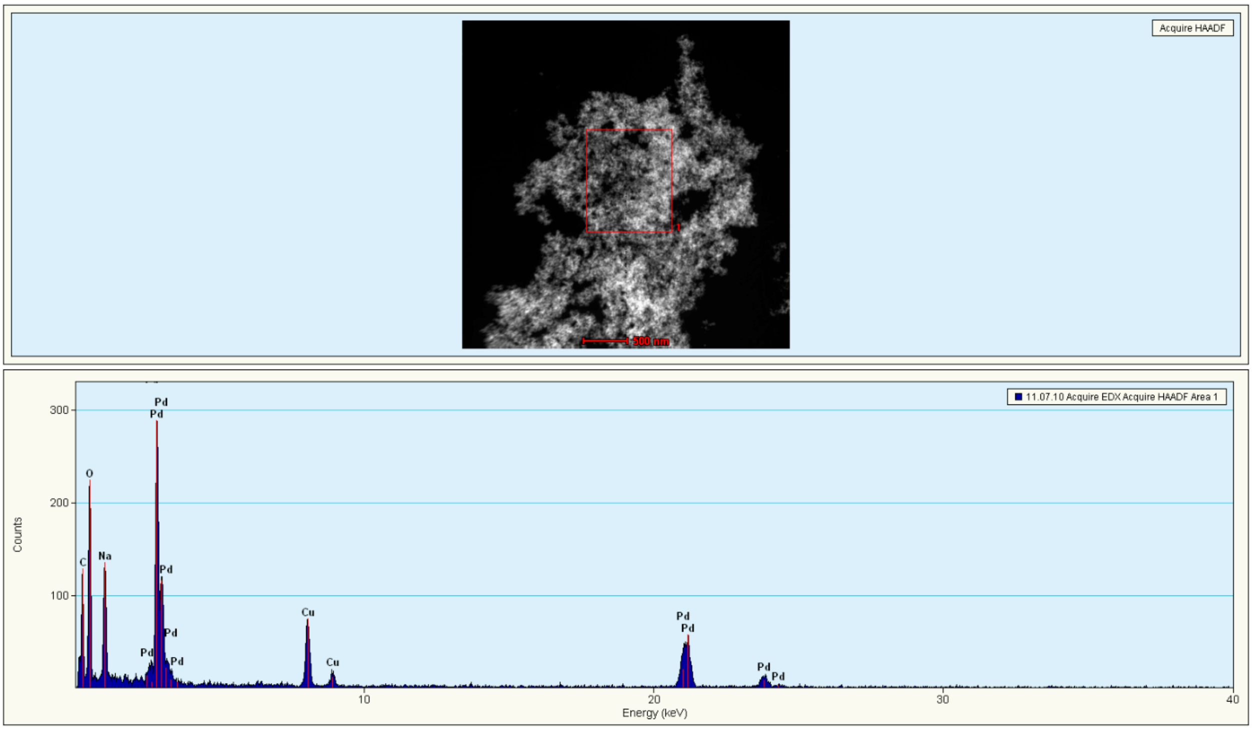

Figure S5. TEM image of Pd NP and its energy-dispersive X-ray spectroscopy (EDX) analysis. The scale bar is 500 nm.

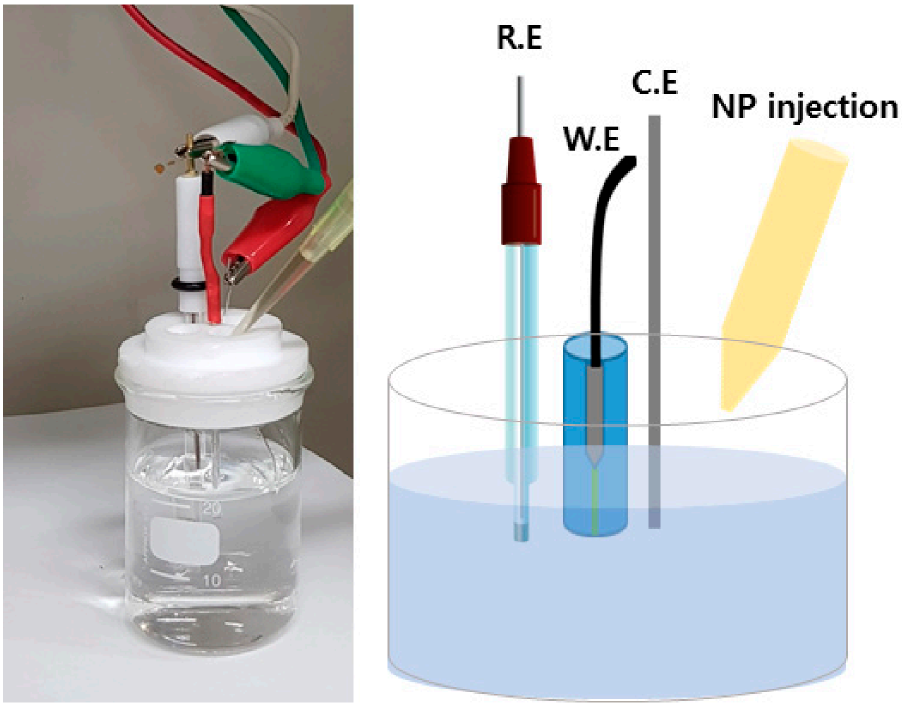

Figure S6. Photo of electrochemical set-up and its schematic illustration.
